# Exploring Better Links Between Clinics and Communities to Improve Population Health

**DOI:** 10.5888/pcd12.140568

**Published:** 2015-01-08

**Authors:** Peter A. Briss

In this issue of *Preventing Chronic Disease*, Kristal et al (1) report on analyses of several standardized questions about health behaviors related to sugar-sweetened beverage consumption and other aspects of diet and physical activity in a Bronx population served by several federally qualified health centers. These analyses are part of a larger project that links clinical and community approaches to measuring and improving diet and physical activity behaviors. Other components of the larger project included electronic health record (EHR)–based referrals to community diabetes prevention resources and enhanced community access to more healthful foods. The larger project illustrates several innovative approaches that health care and community organizations might use for advancing population health through improved delivery of high-quality health care and better links between clinical and community resources.

The study by Kristal et al highlights the importance of measuring and acting on health-risk behaviors in both clinical and community settings. In the United States, just 4 modifiable risk behaviors (tobacco use, poor nutrition, physical inactivity, and unhealthy alcohol use) account for about 40% of mortality (2). In their study, Kristal et al programmed questions for assessing selected risk behaviors into the EHR. These questions, derived from the state-based Behavioral Risk Factor Surveillance System, also are used in the New York City Community Health Survey. The use of similar questions at several population levels (ie, clinic, community, and state) increases the ability of health care and community health providers and decision makers to use comparable data on risk factors and health outcomes in populations in different settings and at local, state, and national levels for diverse purposes — detecting problems, monitoring trends, targeting and triggering interventions, and comparing the results achieved in various health care and community contexts.

The study by Kristal et al also illustrates how use of the EHR and team-based care can better support disease prevention and health promotion. For many clinical providers, preventive care can be difficult to deliver consistently because of limitations on provider time. To illustrate this problem, one study estimated that in a typical primary care practice with a panel size of 2,500 and an age and sex distribution similar to that of the United States, a physician would require 7.4 hours per day to provide all services recommended by the US Preventive Services Task Force (3). In the study by Kristal et al, the process of documenting lifestyle-behavior questions was incorporated into clinic workflows by automatically generating EHR reminders, prompting nursing staff to ask the questions during intake assessments. Physicians then followed up on the results. This approach to building prevention into clinic workflows and systematically sharing tasks between clinic staff can improve the extent and efficiency of preventive care delivery. As the US health care system continues to shift from paying for services to paying for value, and as quality measures increasingly affect those payments, team-based care will become increasingly important for delivering preventive and clinical services.

A recent Institute of Medicine report noted that “achieving substantial and lasting improvements in population health will require a concerted effort from . . . [a wide range of entities including both health care and public health] . . . aligned with a common goal” (4). The study described by Kristal et al expands the menu of innovative opportunities for bridging clinical settings and communities with the intention of improving population health. Other ongoing efforts operate across a spectrum of populations and settings, ranging from the local to national levels (Box). These efforts underscore the recognition that the health care and public health sectors each has an important role in improving population health and that neither can maximize health by working in isolation.

**Box. Selected Recent Projects That Link Health Care and Community Approaches to Improve Population Health**

**Table:** 

Project	Description
Million Hearts (5)	Million Hearts is a large national effort that aims to prevent 1 million heart attacks and strokes from 2012 to 2017 by making heart-healthy lifestyle choices easier and by improving care for people needing treatment.
The National Diabetes Prevention Program (6)	The National Diabetes Prevention Program links people at high risk of developing diabetes to community-delivered, evidence-based lifestyle interventions that can greatly reduce their risk of developing diabetes and, because it is delivered by lay people in community settings, can be more convenient and cost-effective than similar interventions delivered in health care settings.
Partnership for a Healthy Durham (7)	The Partnership for a Healthy Durham has pulled together many stakeholders to improve health among the most vulnerable residents of Durham County, North Carolina. The project has expanded over time from interventions to improve access to high-quality health care to include environmental approaches to promoting physical activity and efforts to improve primary education.
Truman Medical Centers Healthy Harvest Produce Market (8)	Truman Medical Centers, an acute care hospital system located in an urban food desert, has established a farmers market to enhance access to fresh and healthful fruits and vegetables for its patients and staff.
